# Supplementary material for: Ethanol Extract of Campsis grandiflora Flower and Its Organic Acid Components Have Inhibitory Effects on Autoinducer Type 1 Quorum Sensing
Source: Molecules. 2020 Oct 15;25(20):4727. doi: 10.3390/molecules25204727 (PMC7587560; doi:10.3390/molecules25204727)
Supplement: Supplementary file 1 [file molecules-25-04727-s001.pdf]

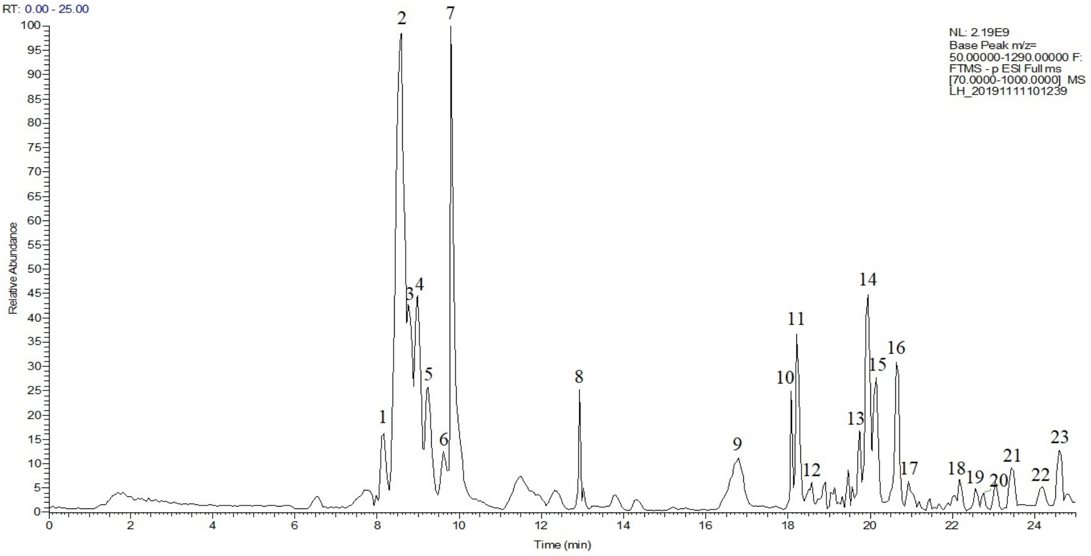

The total ion current of CFP identified by UHPLC-HESI-MS/MS

Peak 1

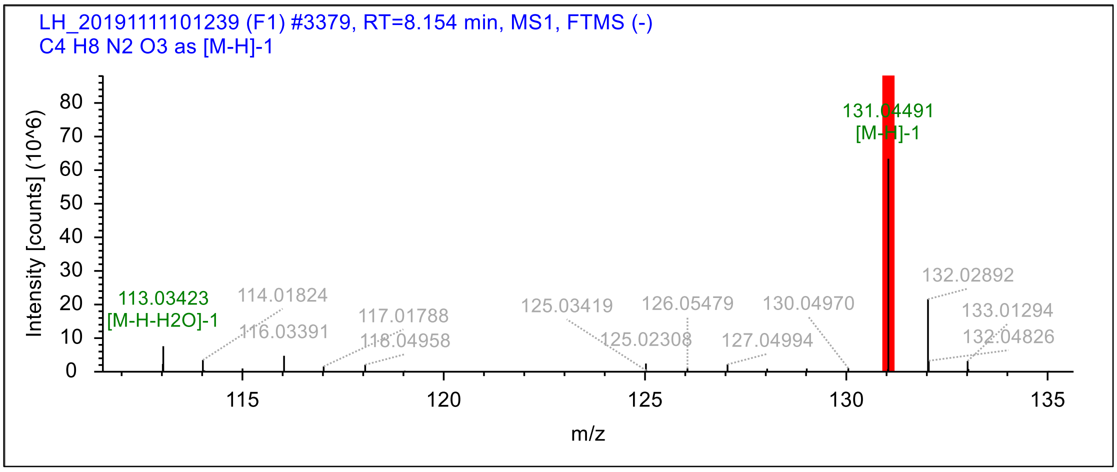
LH_20191111101239 (F1) #3379, RT=8.154 min, MS1, FTMS (-)
C4 H8 N2 O3 as [M-H]-1
131.04491
[M-H]-1
113.03423
[M-H-H2O]-1
114.01824
116.03391
117.01788
118.04958
125.03419
125.02308
126.05479
127.04994
130.04970
132.02892
133.01294
132.04826
Intensity [counts] (10^6)
m/z

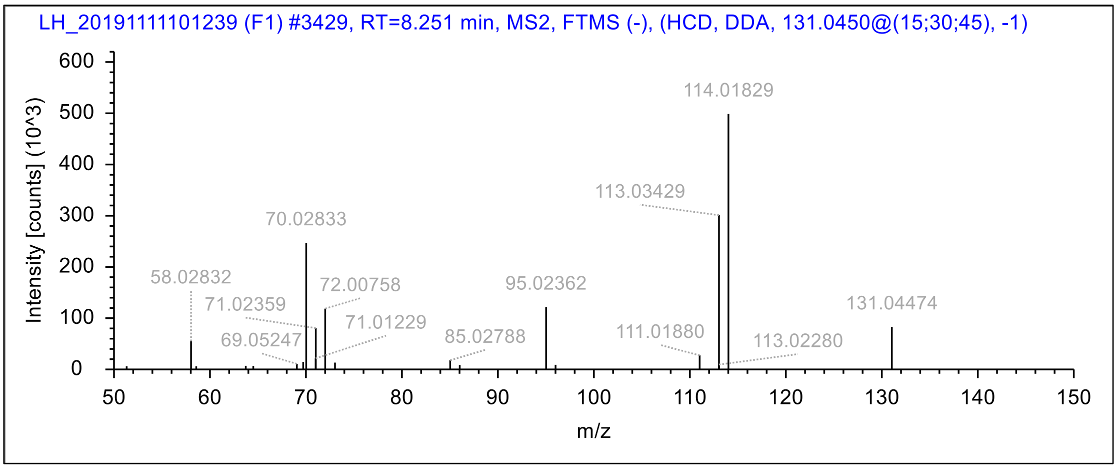
LH_20191111101239 (F1) #3429, RT=8.251 min, MS2, FTMS (-), (HCD, DDA, 131.0450@(15;30;45), -1)
114.01829
113.03429
70.02833
58.02832
71.02359
72.00758
71.01229
69.05247
95.02362
85.02788
111.01880
113.02280
131.04474
Intensity [counts] (10^3)
m/z

Peak 2

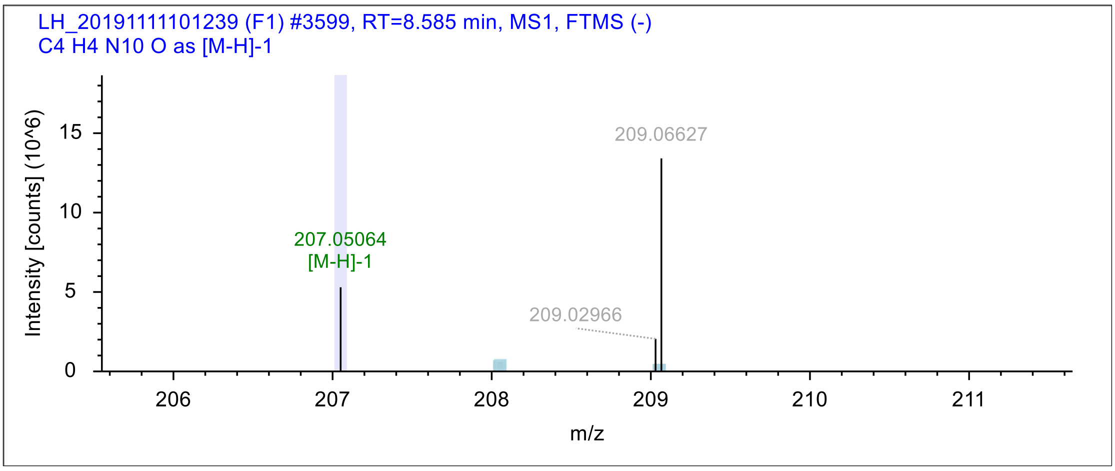
LH_20191111101239 (F1) #3599, RT=8.585 min, MS1, FTMS (-)
C4 H4 N10 O as [M-H]-1
209.06627
207.05064
[M-H]-1
209.02966
Intensity [counts] (10^6)
m/z

Peak 3

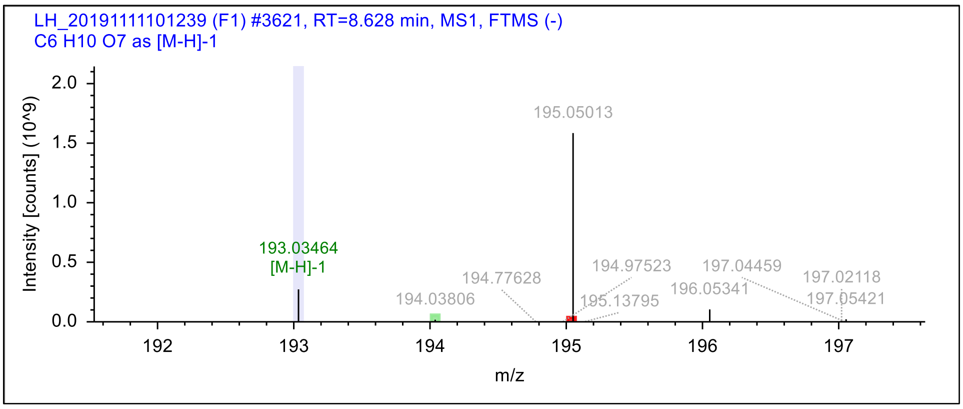

LH_20191111101239 (F1) #3621, RT=8.628 min, MS1, FTMS (-)
C6 H10 O7 as [M-H]-1
Intensity [counts] (10^9)
193.03464
[M-H]-1
195.05013
194.77628
194.03806
194.97523
195.13795
197.04459
196.05341
197.02118
197.05421
m/z

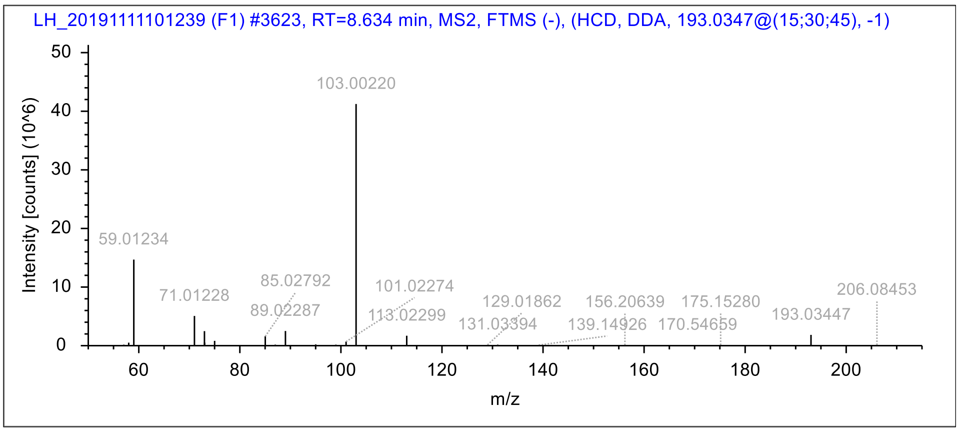

LH_20191111101239 (F1) #3623, RT=8.634 min, MS2, FTMS (-), (HCD, DDA, 193.0347@(15;30;45), -1)
Intensity [counts] (10^6)
103.00220
59.01234
71.01228
85.02792
89.02287
101.02274
113.02299
129.01862
131.03394
156.20639
139.14926
175.15280
170.54659
193.03447
206.08453
m/z

Peak 4

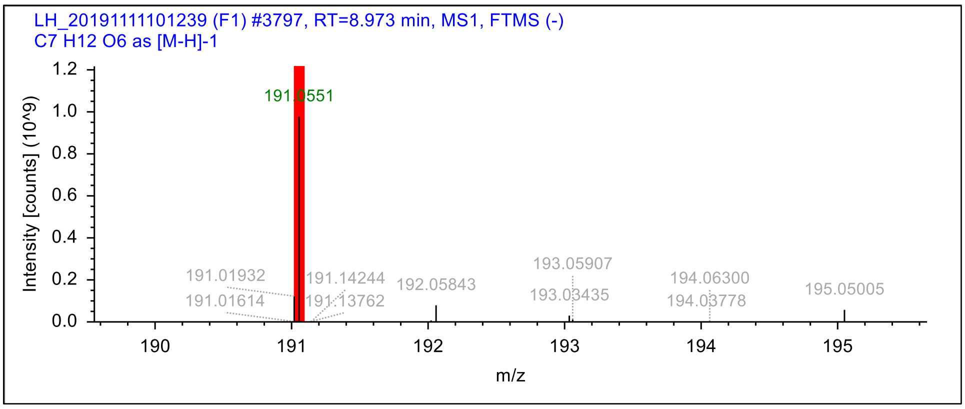

LH_20191111101239 (F1) #3797, RT=8.973 min, MS1, FTMS (-)
C7 H12 O6 as [M-H]-1
Intensity [counts] (10^9)
191.01932
191.01614
191.14244
191.13762
192.05843
193.05907
193.03435
194.06300
194.03778
195.05005
m/z

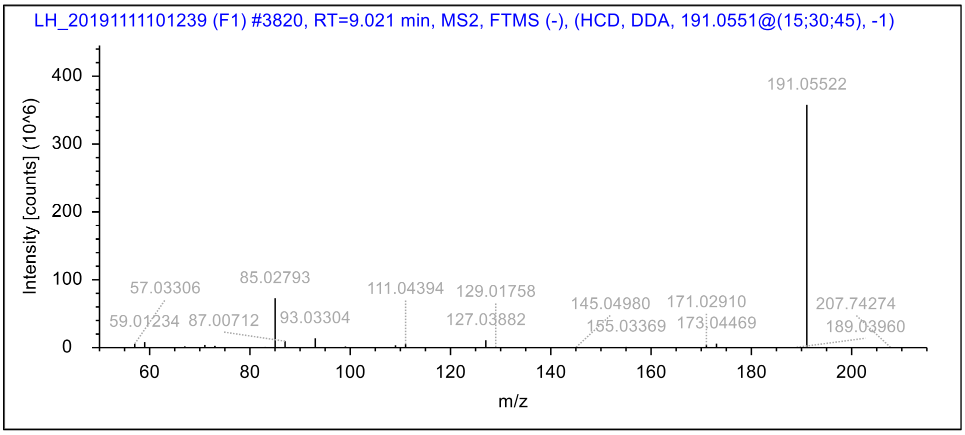

LH_20191111101239 (F1) #3820, RT=9.021 min, MS2, FTMS (-), (HCD, DDA, 191.0551@(15;30;45), -1)
Intensity [counts] (10^6)
191.05522
57.03306
59.01234
85.02793
87.00712
93.03304
111.04394
129.01758
127.03882
145.04980
155.03369
171.02910
173.04469
207.74274
189.03960
m/z

Peak 5

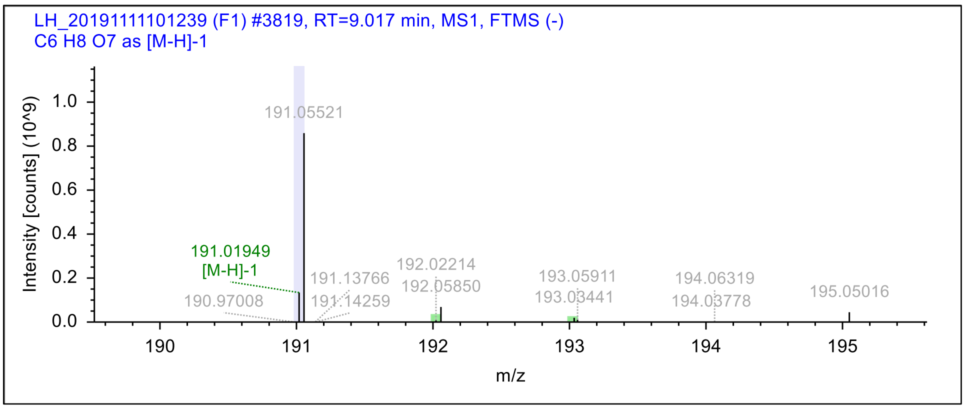
LH_20191111101239 (F1) #3819, RT=9.017 min, MS1, FTMS (-)
C6 H8 O7 as [M-H]-1
Intensity [counts] (10^9)
191.05521
191.01949
[M-H]-1
190.97008
191.13766
191.14259
192.02214
192.05850
193.05911
193.03441
194.06319
194.03778
195.05016
m/z

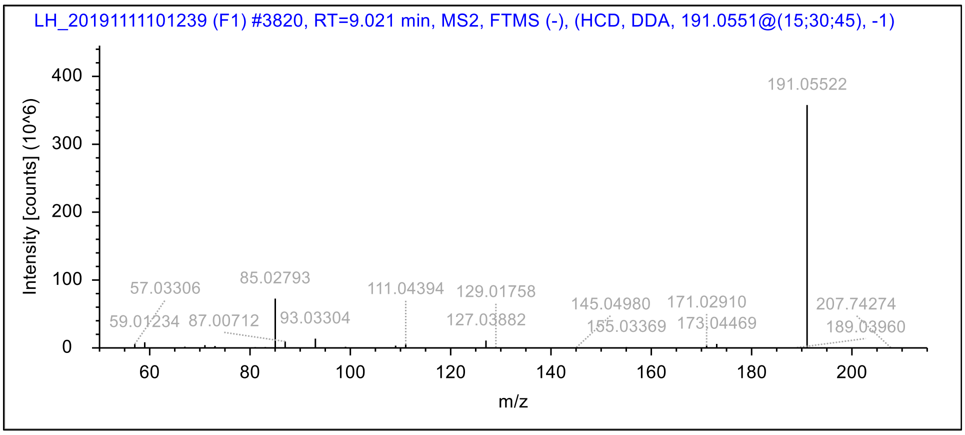
LH_20191111101239 (F1) #3820, RT=9.021 min, MS2, FTMS (-), (HCD, DDA, 191.0551@(15;30;45), -1)
Intensity [counts] (10^6)
191.05522
57.03306
59.01234
85.02793
87.00712
93.03304
111.04394
129.01758
127.03882
145.04980
155.03369
171.02910
173.04469
207.74274
189.03960
m/z

Peak 6

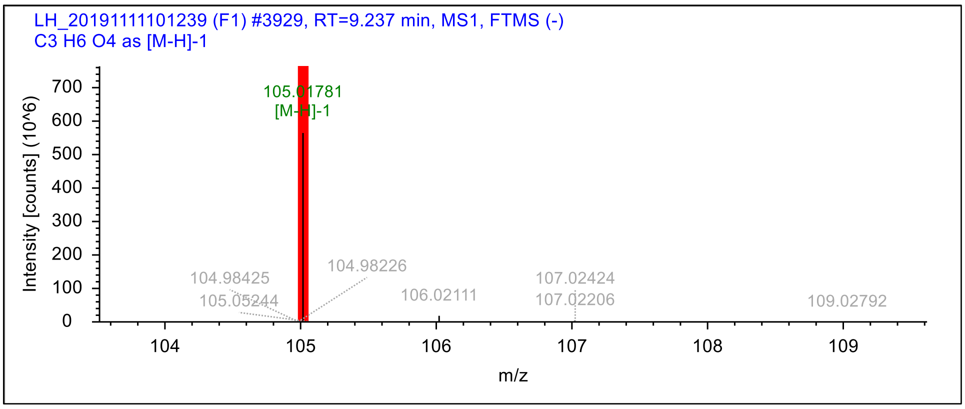
LH_20191111101239 (F1) #3929, RT=9.237 min, MS1, FTMS (-)
C3 H6 O4 as [M-H]-1
Intensity [counts] (10^6)
105.01781
[M-H]-1
104.98425
105.05244
104.98226
106.02111
107.02424
107.02206
109.02792
m/z

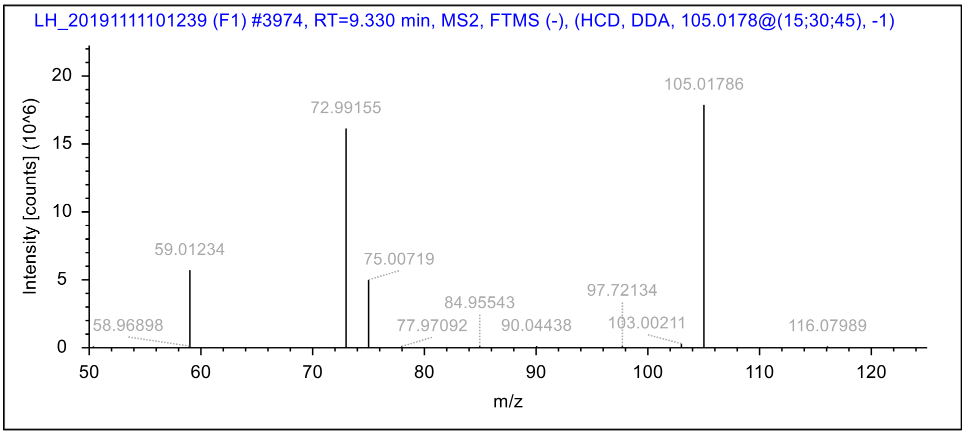
LH_20191111101239 (F1) #3974, RT=9.330 min, MS2, FTMS (-), (HCD, DDA, 105.0178@(15;30;45), -1)
Intensity [counts] (10^6)
105.01786
72.99155
59.01234
75.00719
58.96898
77.97092
84.95543
90.04438
97.72134
103.00211
116.07989
m/z

Peak 7

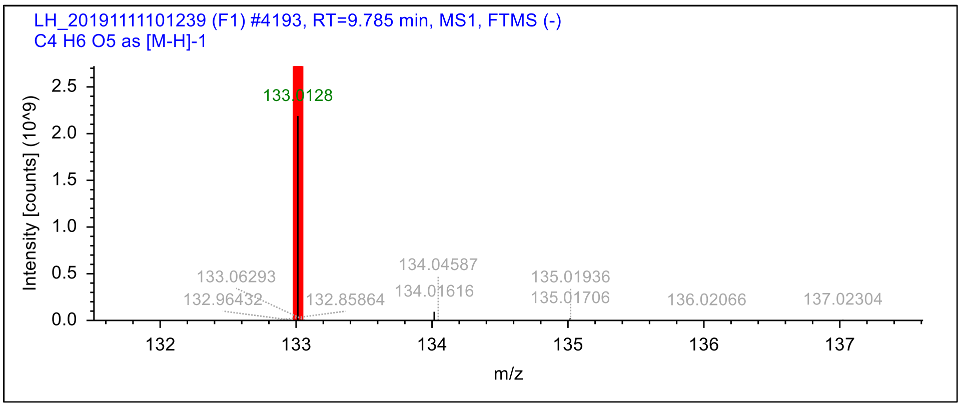
LH_20191111101239 (F1) #4193, RT=9.785 min, MS1, FTMS (-)
C4 H6 O5 as [M-H]-1
133.0128
133.06293
132.96432
132.85864
134.04587
134.01616
135.01936
135.01706
136.02066
137.02304
Intensity [counts] (10^9)
m/z

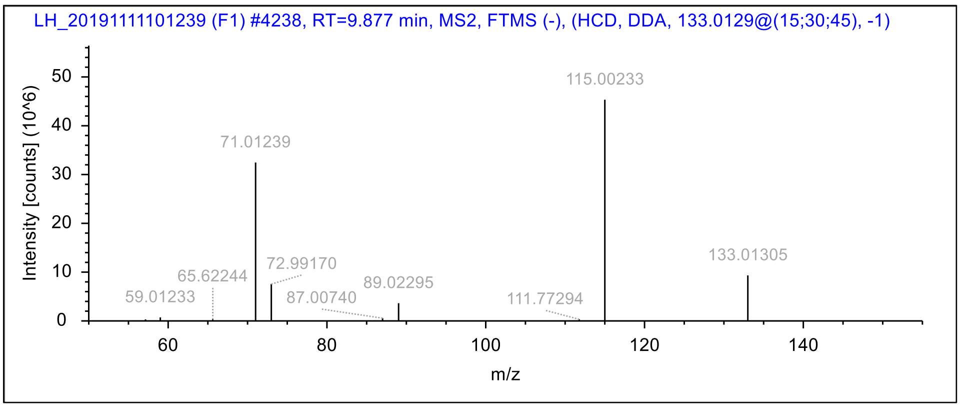
LH_20191111101239 (F1) #4238, RT=9.877 min, MS2, FTMS (-), (HCD, DDA, 133.0129@(15;30;45), -1)
115.00233
71.01239
133.01305
72.99170
65.62244
89.02295
87.00740
59.01233
111.77294
Intensity [counts] (10^6)
m/z

Peak 8

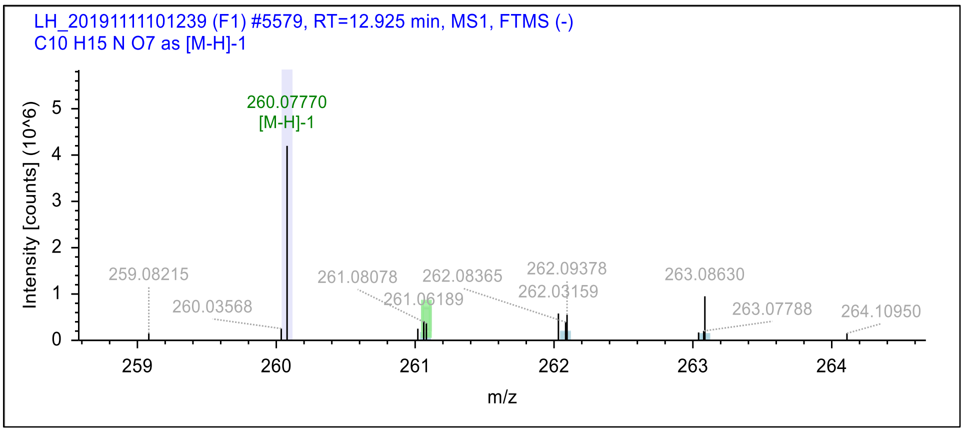
LH_20191111101239 (F1) #5579, RT=12.925 min, MS1, FTMS (-)
C10 H15 N O7 as [M-H]-1
260.07770
[M-H]-1
259.08215
260.03568
261.08078
261.06189
262.08365
262.09378
262.03159
263.08630
263.07788
264.10950
Intensity [counts] (10^6)
m/z

Peak 9

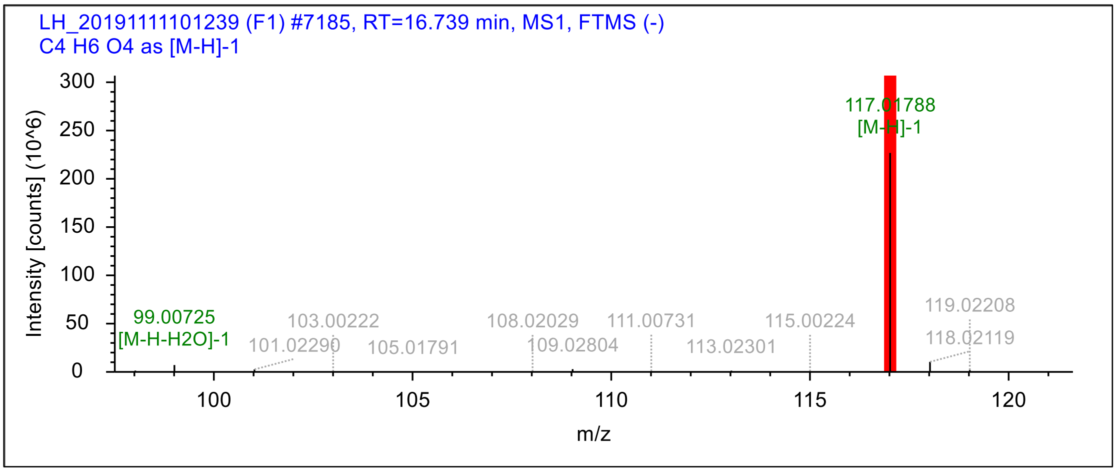

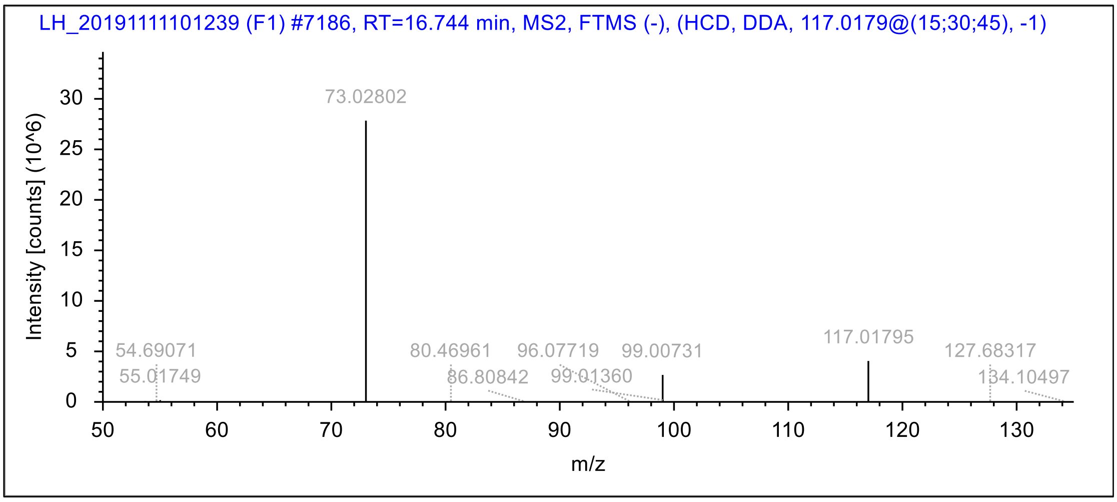

Peak 10

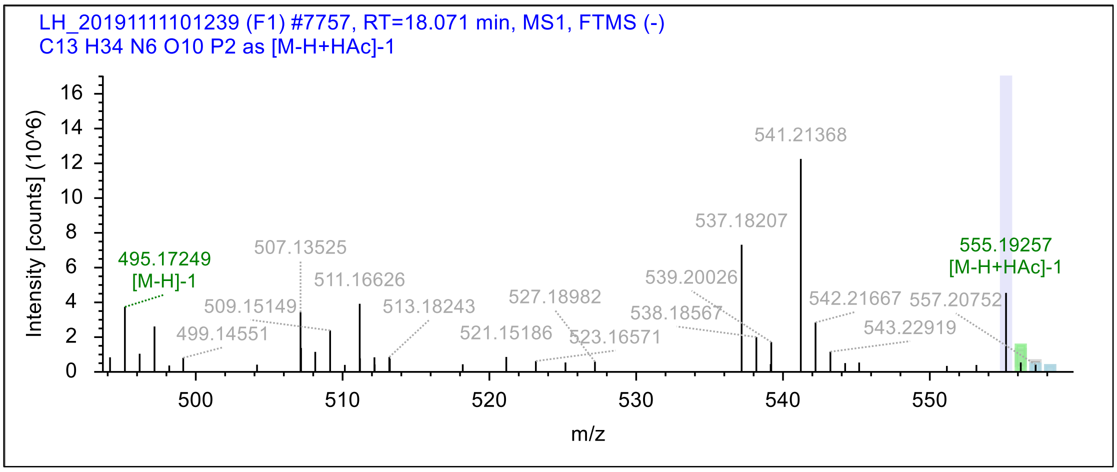

Peak 11

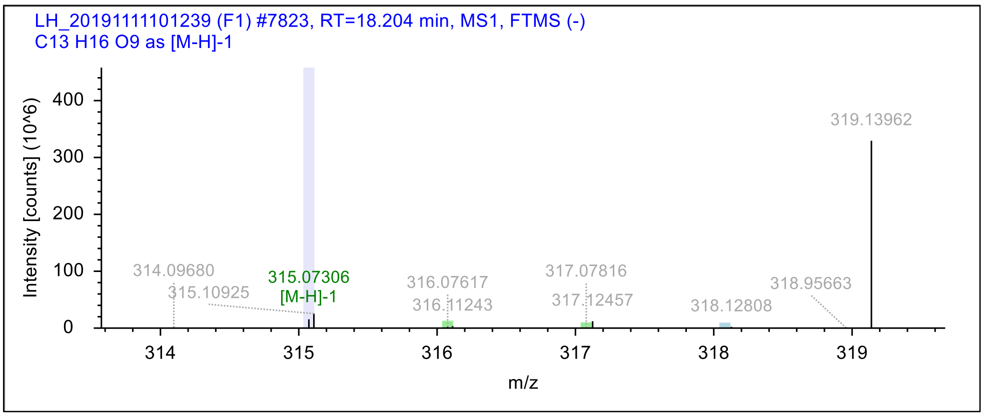

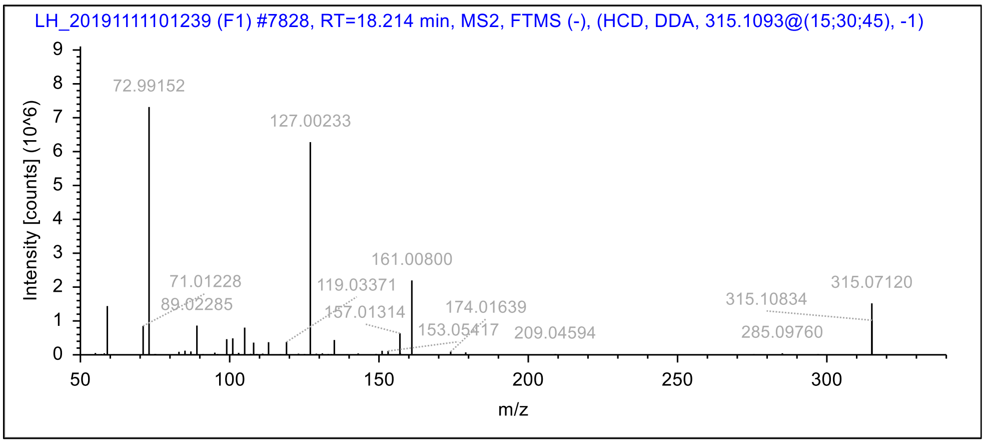

Peak 12

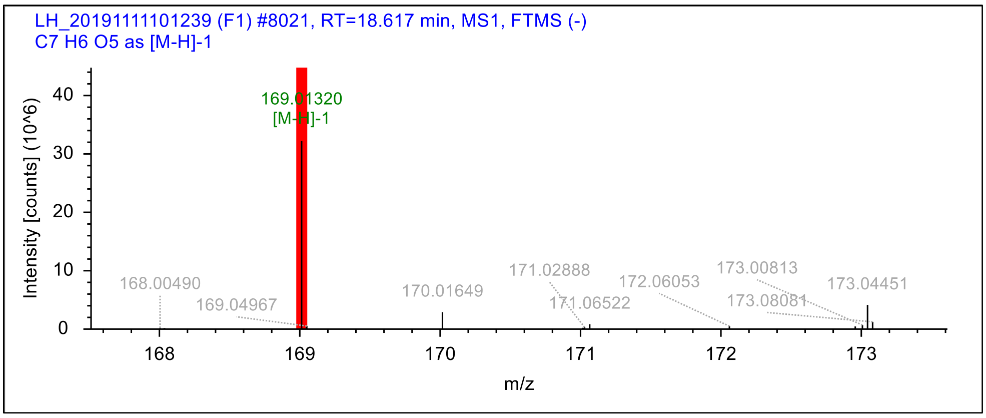

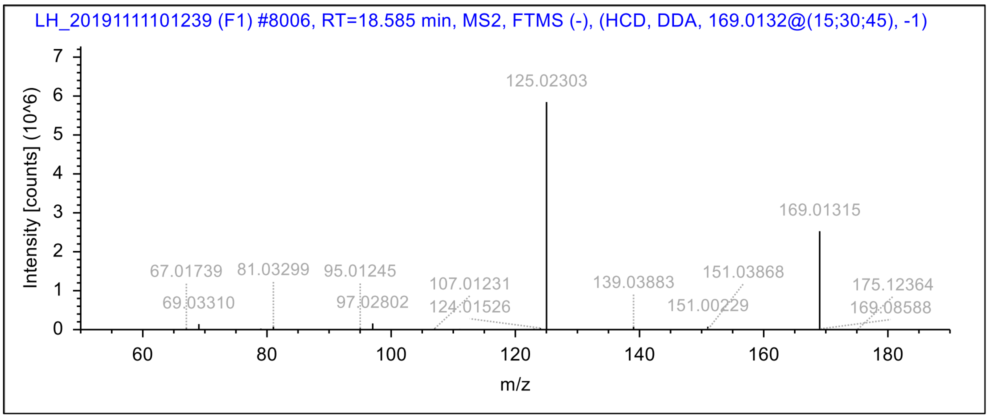

Peak 13

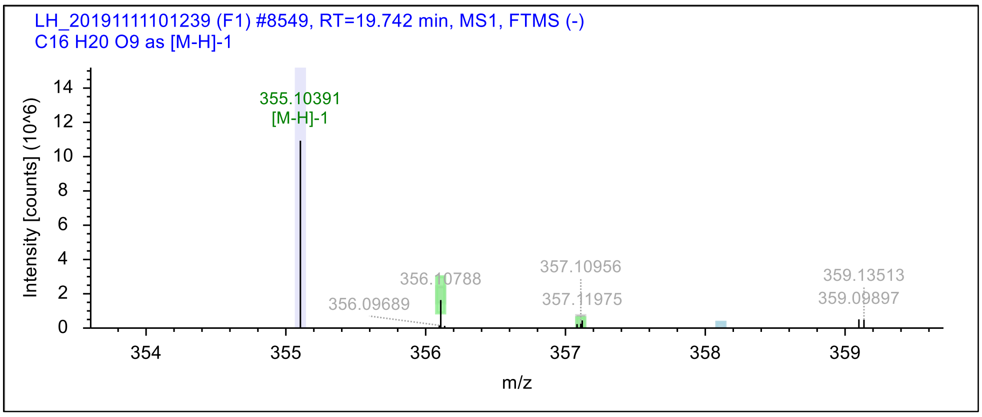

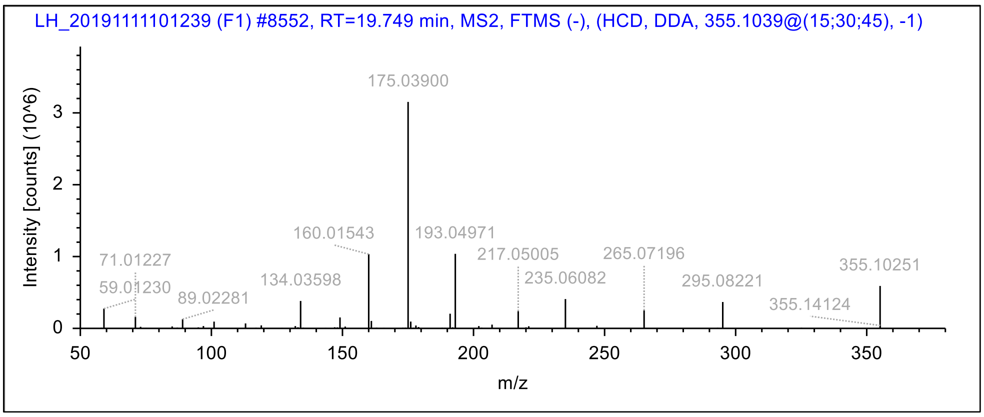

Peak 14

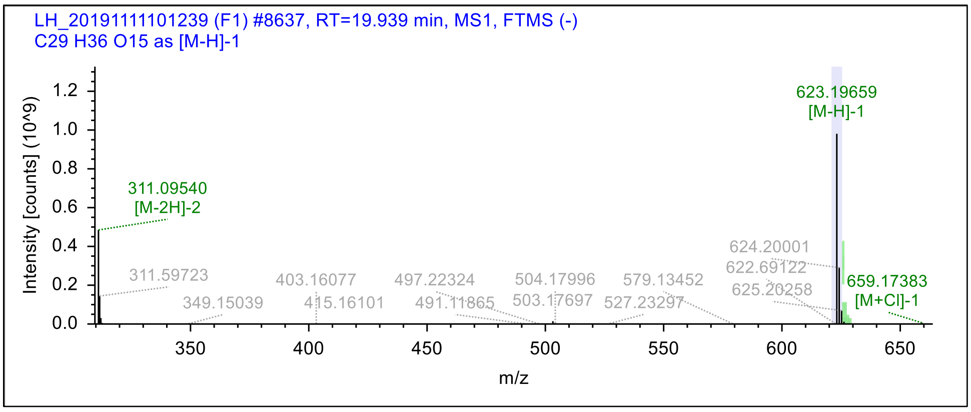

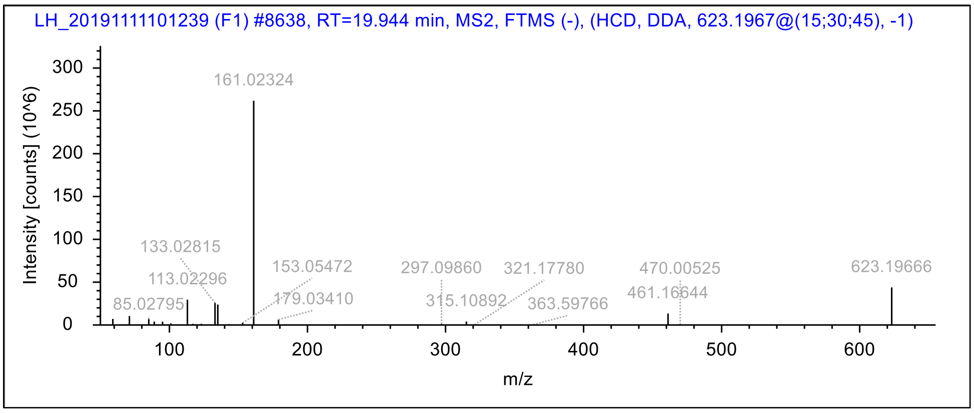

Peak 15

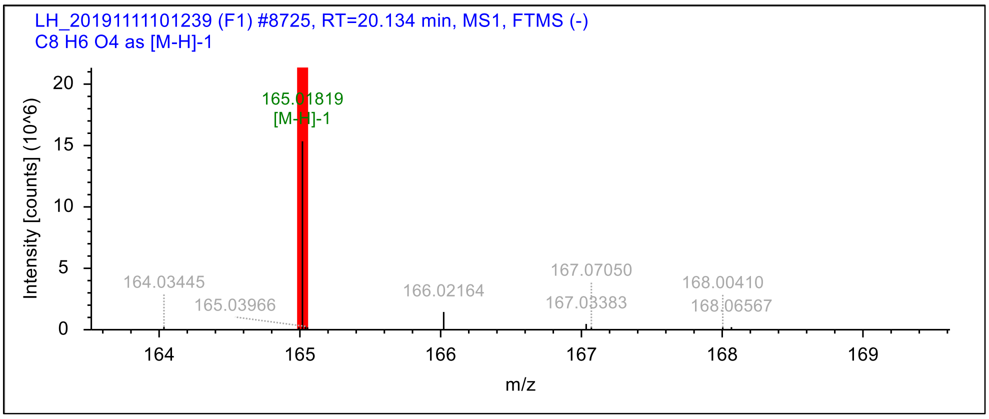

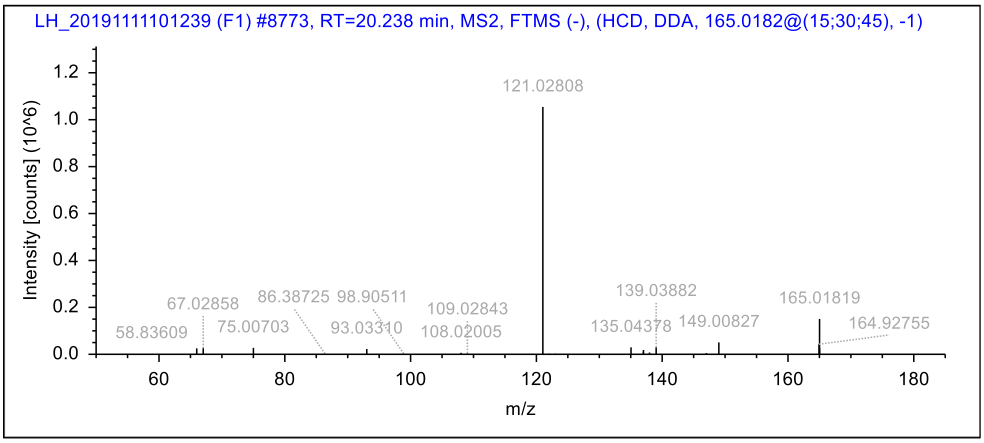

Peak 16

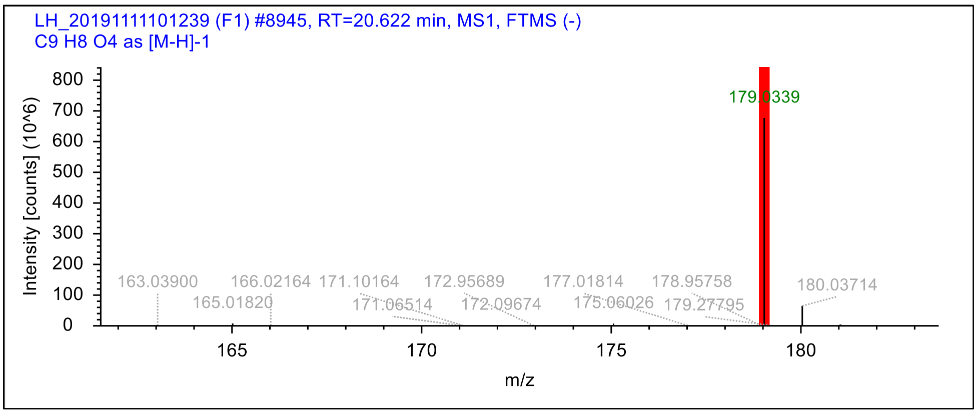

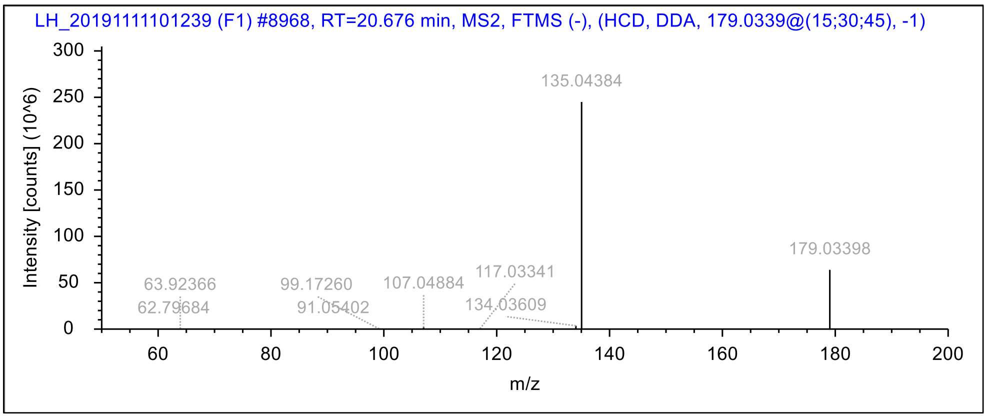

Peak 17

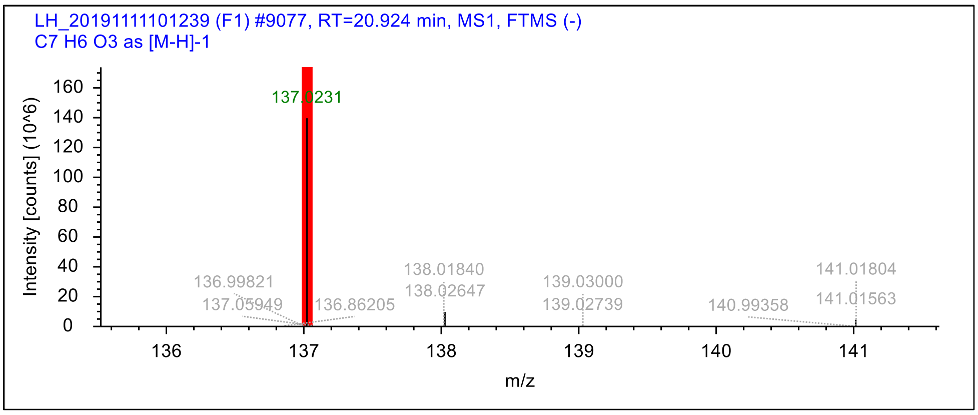

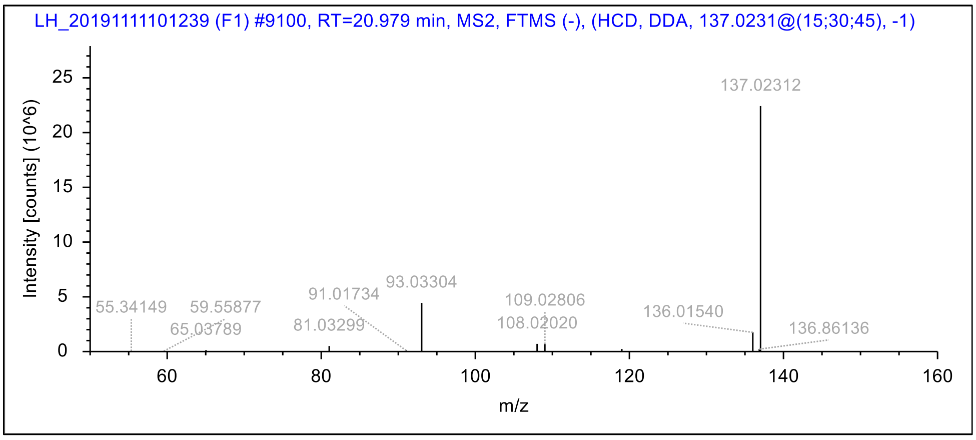

Peak 18

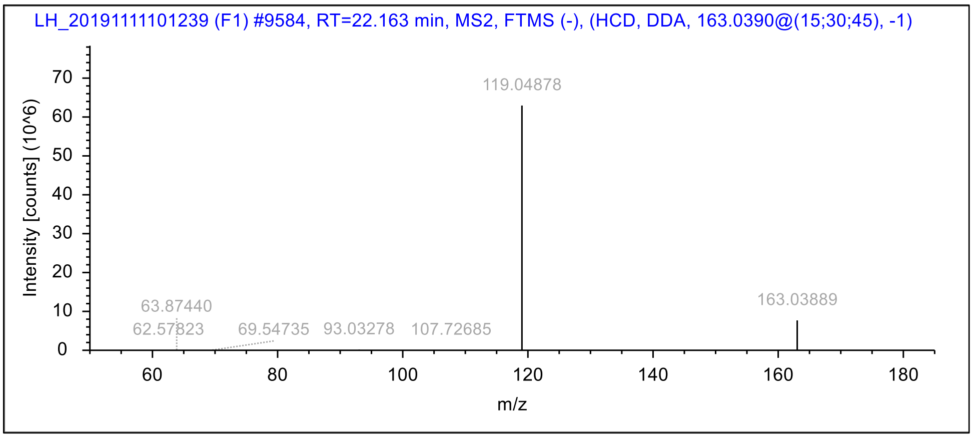

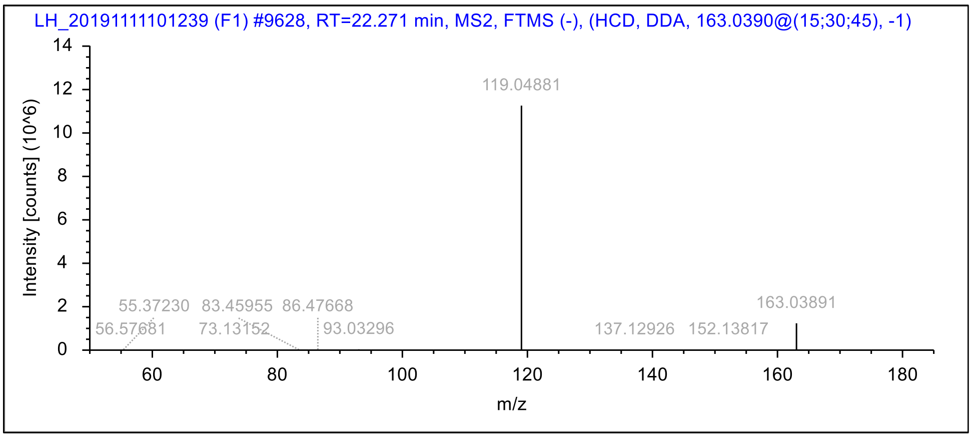

Prak 19

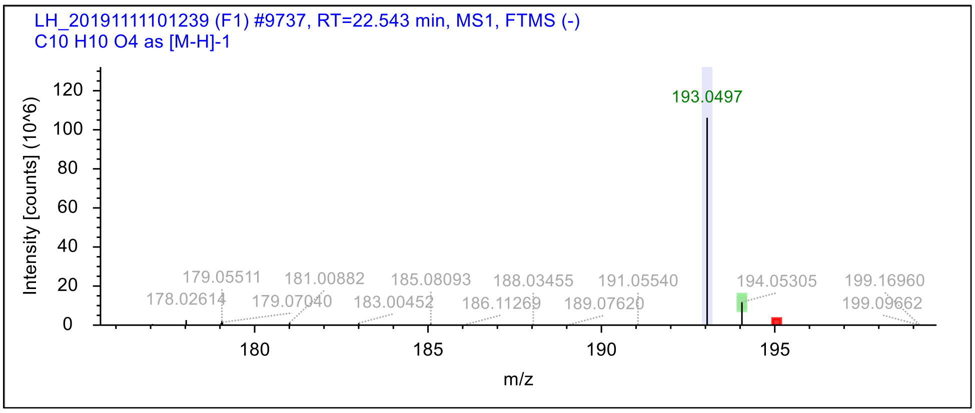

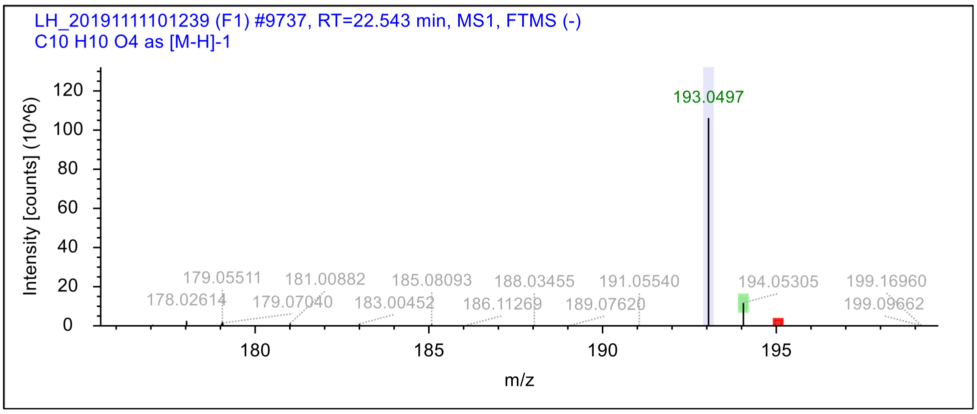

Peak 20

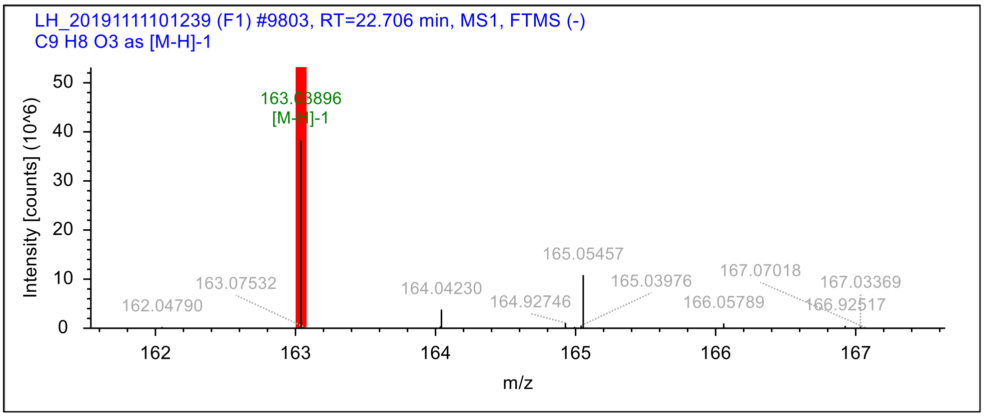

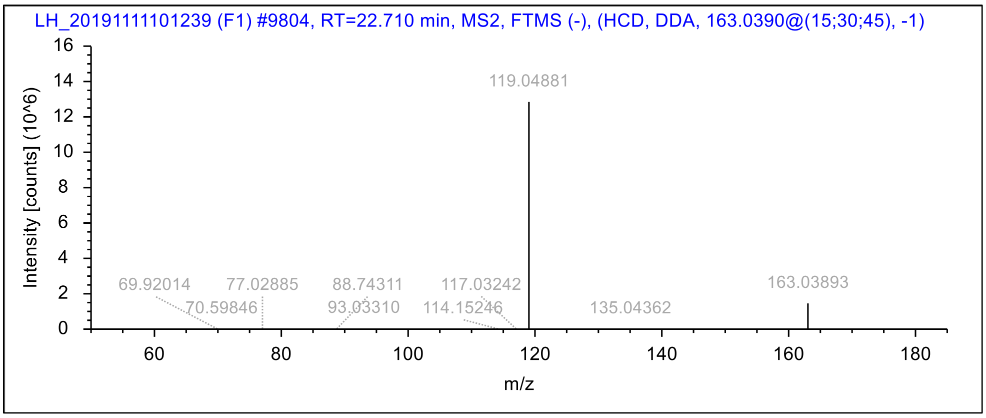

Peak 21

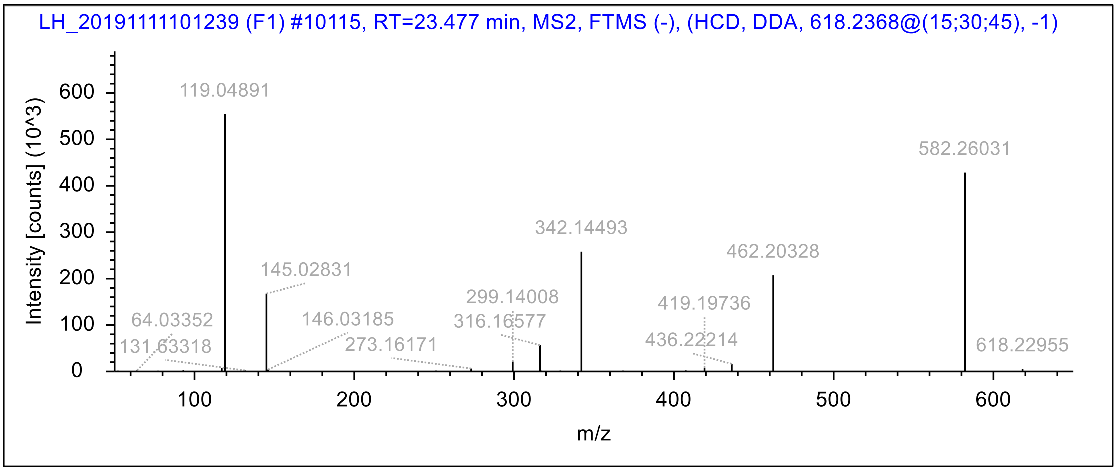

Peak 22

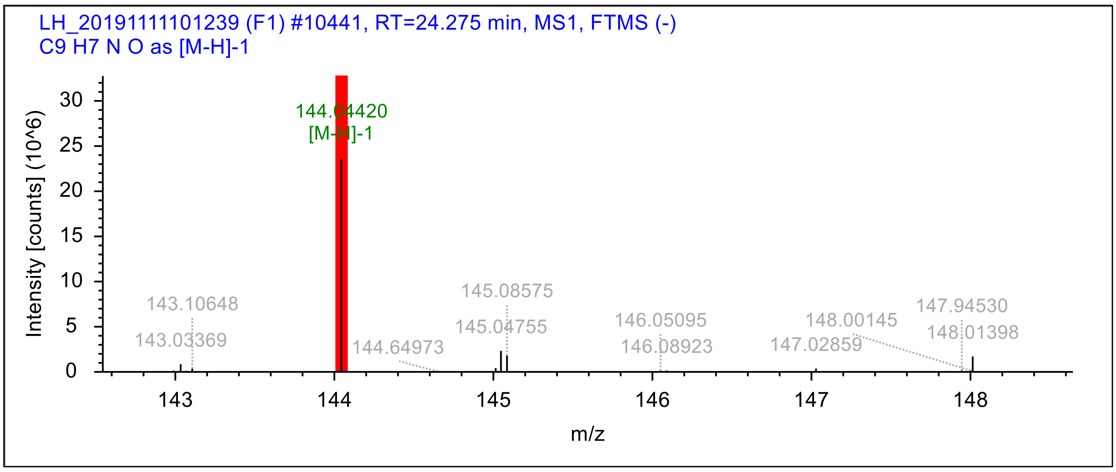

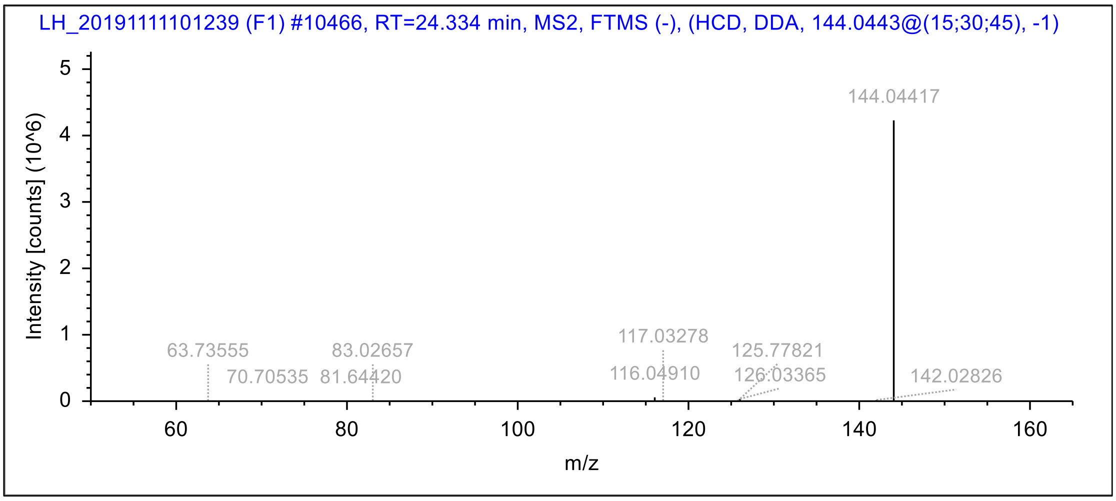

Peak 23

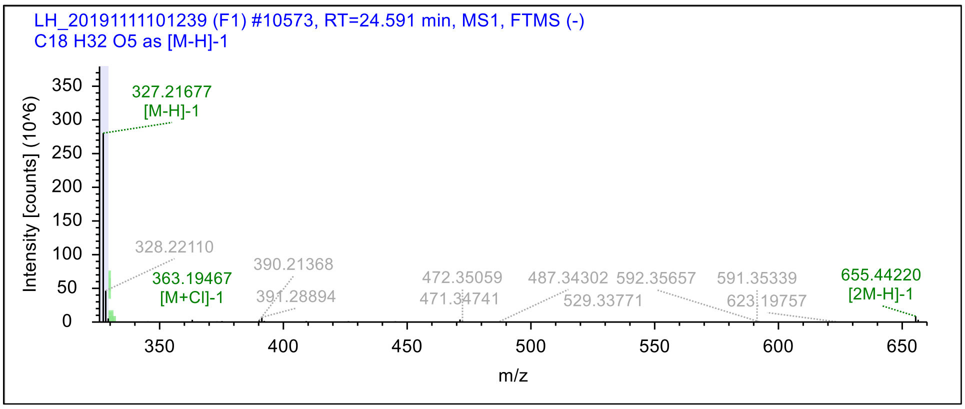

LH_20191111101239 (F1) #10573, RT=24.591 min, MS1, FTMS (-)
C18 H32 O5 as [M-H]-1
327.21677
[M-H]-1
328.22110
363.19467
[M+Cl]-1
390.21368
391.28894
472.35059
471.34741
487.34302
529.33771
592.35657
591.35339
623.19757
655.44220
[2M-H]-1
Intensity [counts] (10^6)
m/z

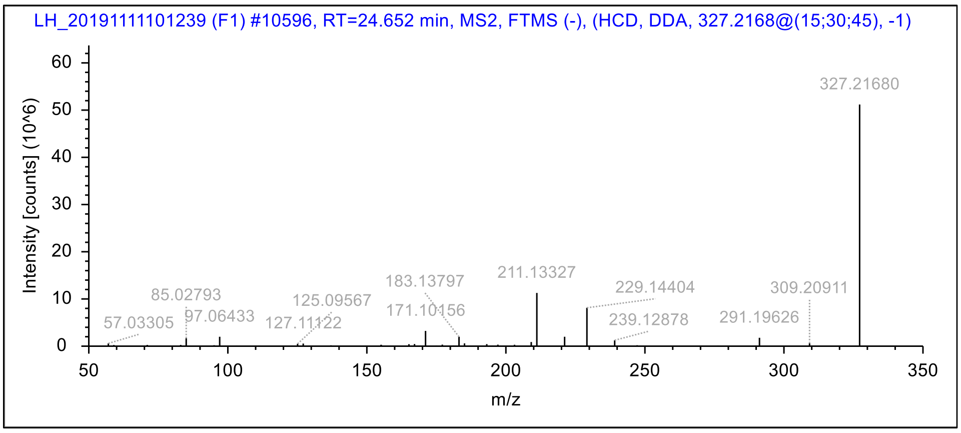

LH_20191111101239 (F1) #10596, RT=24.652 min, MS2, FTMS (-), (HCD, DDA, 327.2168@(15;30;45), -1)
327.21680
85.02793
57.03305
97.06433
125.09567
127.11122
183.13797
171.10156
211.13327
229.14404
239.12878
309.20911
291.19626
Intensity [counts] (10^6)
m/z
